# Ethnopharmacology, phytochemistry, pharmacology and toxicity of the genus *Gouania*

**DOI:** 10.1016/j.heliyon.2024.e40933

**Published:** 2024-12-05

**Authors:** Hannington Gumisiriza, Eunice Apio Olet, Lydia Mwikali, Racheal Akatuhebwa, Owen Kembabazi, Timothy Omara, Julius Bunny Lejju

**Affiliations:** a Department of Chemistry, Mbarara University of Science and Technology, P.O. Box 1410, Mbarara, Uganda; b Department of Biology, Mbarara University of Science and Technology, P.O. Box 1410, Mbarara, Uganda; c Department of Agriculture, Agribusiness, and Environment, Bishop Stuart University, P.O. Box 09, Mbarara, Uganda; d Department of Marketing and International Business, Makerere University Business School, P.O. Box 1337, Kampala, Uganda; e Department of Chemistry, College of Natural Sciences, Makerere University, P.O. Box 7062, Kampala, Uganda

**Keywords:** Gouaniasides, Gouanogenin, Gouanic acids, *Gouania longipetala*, Acute toxicity

## Abstract

The genus *Gouania* (Rhamnaceae) comprises at least 50 recognized species distributed across tropical and subtropical regions. *Gouania* species have been ethnomedicinally used to treat a variety of ailments. Despite their widespread medicinal use, there is no comprehensive documentation that consolidates the ethnobotanical knowledge, phytochemicals, pharmacological properties, and toxicity of *Gouania* species. Herein, this review details the ethnopharmacology, phytochemical constituents, pharmacological properties and toxicity of *Gouania* species to provide perspectives for future research on this genus. Based on available literature, herbal preparations from *Gouania* species have been used to treat ailments related to the digestive, cardiovascular, respiratory, skin, musculoskeletal, reproductive, endocrine and urological systems. Extracts and isolated compounds from seven *Gouania* species (*G. leptostachya*, *G. longipetala*, *G. lupulozdes*, *G. macrocarpa*, *G. longispicata*, *G. obtusifolia*, and *G. ulmifolia*) have demonstrated promising anticancer, antimicrobial, antioxidant, and antiviral properties, supporting their ethnomedicinal uses. To date, 64 compounds (including 6 phenolic compounds, 24 flavonoids, and 34 terpenoids) have been isolated and characterized in the genus mainly as gouaniasides I-IX, gouanogenins, and gouanic acids. Most *Gouania* species remain unexplored for their potential bioactivities. The identification of more than 54 % as novel compounds from just seven *Gouania* species highlights the genus as a promising source for discovering new therapeutic agents to combat the growing challenge of multidrug-resistant pathogens. Conducting extensive phytochemical and pharmacological analyses across a broader array of *Gouania* species could unveil a more comprehensive profile of bioactive compounds, and pave way for innovative treatments against a diverse range of pathogens and diseases.

## Highlights

• The ethnomedical uses of the genus *Gouania* are extensive.
• Only seven *Gouania* species have been scientifically explored.
• Of the 64 pure compounds isolated from seven *Gouania* species, over 54 % are novel.
• *Gouania* genus offers potential for discovering novel therapeutics to combat the growing range of pathogens.

## 1 Introduction

The *Gouania* Jacq. genus comprises of up to 70 distinct taxa in the buckthorn family (Rhamnaceae) [1]. The genus was established in 1763 by Jacquin based on two species from Haiti ("Domingensibus") namely: *G. tomentosa* and *G. glabra* [2,3]. The genus name commemorates Antoine Goüan (1733–1821), a pioneer in Linnaean taxonomy [4]. *Gouania* was classified as monophyletic within the tribe *Gouanieae* by Endlicher [5], a classification which was later reconfirmed by Richardson [6]. These plants are characterized as evergreen woody vines or climbing shrubs [7], with distinctive features that sets them apart from other genera in the family [8,9]. The plants feature alternate leaves, typically serrated with glandular teeth, and small persistent stipules, while the (bisexual) flowers are actinomorphic and arranged in spikes, racemes, or panicles. The flowers are characterized by bell-shaped hypanthium and yellowish-green to whitish hood-shaped petals, with stamens often hooded by petals and arranged around a cup-shaped nectary disk. The ovary is inferior, with 3 locules each containing a single ovule while the fruit is a three-winged schizocarp, splitting into three indehiscent sections with shiny obovate seeds [3].

*Gouania* species are native to tropical zones of Africa (including Madagascar), Australia, Hawaii, Indian Ocean Islands, and Southern Asia [6,8,10,11], Mediterranean and Western Pacific [12]. A revision by Cahen [7] recognized five *Gouania* species in Philippines and Sundaland basing on morphological evidences (including a newly described species, *G. longipedunculata*). Another study reported that two species (*G. leptostachya* and *G. maderaspatana*) are endemic to India [13]. A taxonomic revision of the genus *Gouania* for Madagascar and the other Western Indian Ocean Islands recognized 17 distinct species, of which nine (9) were newly described [4]. Seventeen *Gouania* species were reported in Brazil, out of which only *Gouania ulmifolia* Hook. & Arn. grows in Rio Grande do Sul, but is also found in Southern Brazil, Uruguay and Argentina [14]. Since the last two decades, some *Gouania* species were cited to be critically endangered [8], which calls for establishment of conservation strategies and policies towards sustainability of such species. For instance, a single-island endemic *Gouania meyenii* discovered on Kauai in 1991 by Lorence & Flynn was last observed in 1994, and concerted efforts to relocate it were unsuccessful until the plants were rediscovered in 2020 by drone survey in Kalalau Valley, where conservation collections have been made [15].

*Gouania* species hold significant ecological and medicinal importance in their distributional ranges. They contribute to the biodiversity of ecosystems by providing habitat and food for animals, maintaining soil stability and preventing soil erosion. Members of this genus are renowned for their extensive bioactivities and thus use in ethnomedicine [16]. *Gouania* species feature prominently in traditional medicine where they are used to address cardiovascular, musculoskeletal, respiratory, skin, endocrine, urological, genital and digestive disorders [14,[16], [17], [18], [19], [20], [21]]. *Gouania* species have appreciable antimicrobial [14,22], anti-inflammatory [23], antidiabetic, antilipidemic, antioxidant [18], and estrogenic effects [20] among other bioactivities.

The extensive ethnomedicinal applications of the genus *Gouania* is not surprising given its status as a promising source of novel constituents, with compounds like gouanogenins, gouaniasides, and gouanic acids, which are named after the genus [13,14,[24], [25], [26]]. Triterpenes are reported to be the most abundant phytochemicals in the *Gouania* genus, followed by flavonoids [25,[27], [28], [29]]. Studies on these compounds have exhibited diverse bioactivities including antioxidant, neuraminidase inhibition, anticancer, and antibacterial effects [25,27,28]. Despite their widespread medicinal use, there is no comprehensive documentation that consolidates the ethnobotanical knowledge, phytochemistry, pharmacology, and toxicity of the genus. Herein, this review collated literature on the ethnopharmacology, phytochemical constituents, pharmacological properties and toxicity of *Gouania* species to provide perspectives for future research on this genus.

## 2 Ethnomedicinal uses of *Gouania* species

*Gouania* species are used to treat up to 84 ailments, encompassing conditions affecting the digestive, cardiovascular, respiratory, skin, musculoskeletal, reproductive, endocrine and urological systems (Table 1). As an example, *G. longipetala* aerial parts (stems and leaves) are used in traditional medicine for the treatment of a variety ailments such as swelling, pain, gout, lumbago, venereal diseases, female infertility and cardiovascular diseases [27,30]. In Nigeria, *G. longipetala* has been used as genital stimulant and laxative [18]. The leaves of *G. longispicata* are used in Uganda to treat stomachache [17], opportunistic infections among HIV/AIDS patients [31] and over other 40 ailments including; allergy, and urinary retention [32]. In Ethiopia, *G. longispicata* is used to treat of oral thrush [33], leprosy and leukoderma [34].

**Table 1:** Ethnobotanical uses of *Gouania* species based on published literature.

Species	Disease/conditions treated	Parts used	Preparation and administration	Country	References
*Gouania longispicata* Engl.	Foetal troubles, anorexia, oral thrush, stomachache, increasing strength in children, swelling in groin, boils, ulcers, malaria, allergy, febrile seizures, urinary retention, palpitations, heat rash, mastitis, syphilis, limb pains, syphilis, sweating, tooth decay, wounds, itching eyes, inflammations, colic pain, itching vagina, worms, hypertension, dizziness, asthma, body weakness, lactation insufficiency, typhoid, leprosy, leukoderma, hasten child birth, treat stomachache and malaria, lung and skin cancers, babesiosis and constipation in livestock	Sap, stem exudate, leaves, roots	Infusion taken orally, ointment, tea	Uganda, Tanzania, Rwanda, Cameroon, Ethiopia, Democratic Republic of Congo	[17,[31], [32], [33], [34],[57], [58], [59], [60], [61], [62], [63], [64]]
*Gouania longipetala* Hemsl.	Swelling, diabetes mellitus, gout, venomous stings, women infertility, cardiovascular diseases, snake bites, edema, venereal diseases, lumbago, malaria, pain, febrifuges, wounds, abdominal pain, cleaning of the born baby, body sweats, wounds, abdominal pain, genital stimulant, laxative, eye treatments, pain killer, heart diseases, lumbago, rickets, skin disorders, analgesic, sore eyes, conjunctivitis, iritis, ophthalmia, trachoma, gastro-intestinal problems, gynecological complaints	Stem, leaves, aerial parts, sap	Decoction/infusion taken orally, ointment, eye drops	Cameroon, South East Asia, Nigeria, Ghana	[16,18,20,30,[65], [66], [67], [68], [69]]
*Gouania lupuloides* Urb.	Teeth cleaning, hypertension, malignant ulcers, pneumonia, depurative, diabetes, leishmaniasis	Root, stem, sap, bark	Decoction taken orally, brushing teeth, topical application	Jamaica, Cuba, Costa Rica, Mexico, Ecuador	[24,36,37,40,43,70]
*Gouania leptostachya* DC.	Sores, numbness, stomachache, diarrhea, inflammations, postpartum herbal bath, food supplement, antispasmodic, fainting, leucorrhea	Leaves, stem, root	Poultice, cold infusion or decoction taken orally, bathing, smoke inhalation, potion	India, Indonesia, Thailand, Vietnam, Bhutan, Nepal	[13,41,44,45,[71], [72], [73]]
*Gouania macrocarpa* DC.	Body pain	Root	Cold infusion mixed with rice gruel and taken orally	India	[13]
*Gouania javanica* Miq.	Oral thrush	Stem	Bubbles are blown to the thrush	Philippines	[46]
*Gouania mauritiana* Lam.	Obesity, diabetes, gastroenteritis and inflammations	Leaf, stem	Not reported	France	[21,47]
*Gouania scandens* (Gaertn.) R.B.Drumm. (Synonym: *Gouania tiliifolia* Lam.)	Emmenagogue, diuretic in dropsy	Not reported	Not reported	Mauritius	[48]
*Gouania latifolia* Reissek	Uterine inflammation and back pain	Bast, leaves	Not reported	Brazil	[49]
*Gouania tiliifolia* Lam.	Cough	Leaves	Crush	Madagascar	[50]
*Gouania polygama* (Jacq.) Urb.	Hypertension, skin infections, stomachic, depurative and diuretic properties	Leaves	decoction and fermentation of *G. polygama* with *Smilax domingensis* and *Pimenta dioica* with cane sugar	Cuba, Mexico	[51,52]
*Gouania tiliaefolia* Lam.	Liver disease, wounds, sores, ulcers, fever, headache	Leaves, whole plant	Leaf juice, poultice on sores	India, Bangladesh, Philippines	[53,[74], [75], [76], [77]]
*Gouania meyenii* Steud.	Fever, headache, skin diseases	Not reported	Not reported	Hawaii	[15]

The stems of *G. lupuloides* (Jamaican chewstick) have been used for teeth cleaning among African-Jamaicans for centuries [24,35]. Traditionally, a pencil-size piece of the bitter vine is chewed on one end to cause extensive foam and is then used to brush the teeth and massage the gums. As a result, *G. lupuloides* extracts have been in market as a mouthwash and dentifrice throughout Jamaica [24]. The Negroes grind stems of *G. lupuloides* in water and use it as antiseptic mouthwash or toothbrush [36]. The roots and stems of *G. lupuloides* are used to treat hypertension, while the sap is used to treat athlete's foot [37]. *Gouania lupuloides* is also used in traditional medicine in Cuba [38], Costa Rica [39] and Ecuador [40].

The leaves of *G. leptostachya* are used by the Lepcha (a tribe of Himalayan range living at the North-East corner of India) to make poultices for treating sores [13]. Leaves and stems of *G. leptostachya* are used by the Indonesians for treatment of stomachache and diarrhea [41]. The Mien of Northern Thailand use *G. leptostachya* as a postpartum herbal bath formula and food supplement, and also to treat convulsion in new born, numbness, fainting [42] and inflammations [23]. The rural folks of North Andaman, India burn the roots of *G. leptostachya* and then inhale the smoke to treat fever [43]. Paste made from the leaves of *G. leptostachya* is used to cure sores and inflammation in India [44,45].

**Fig. 1:**
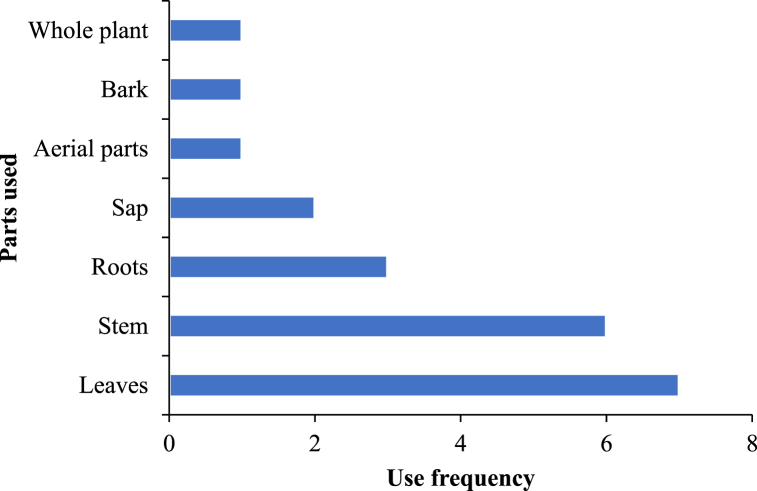
Frequency of use of different parts of *Gouania* species for preparation of herbal remedies.

The stem of *G. javanica* is used to treat fungal infections [46]. *Gouania mauritiana* is traditionally used to reduce cases of obesity and diabetes [21], gastroenteritis and inflammations [47]. *G. scandens* is used as an emmenagogue and diuretic in dropsy [48]. *G. latifolia* is used to treat uterine inflammation and back pain [49]. Leaf decoction of *G. tiliifolia* is orally taken in Madagascar for respiratory disorders [50]. *G. polygama* is used to make a traditional Cuban medicinal refreshment beverage produced by fermentation of a decoction of multispecies plants, chiefly with *Smilax domingensis*, *Pimenta dioica* and cane sugar [51]. *G. polygama* leaf is used to treat skin infections [52]. In India, *Gouania tiliaefolia* is used in Indian ethnomedicine for liver complaints [53], while the infusion from roots of *G. macrocarpa* is consumed to treat body pain [54]. Overall, leaves and stems are the most used parts of species of the *Gouania* genus for treatment of diseases (Fig. 1). The use of leaves may be related to their accessibility and abundance throughout the year, and their principal photosynthetic role and in the storage of therapeutic phytochemicals [55,56].

## 3 Phytochemistry of species in the *Gouania* genus

Several phytochemical screening reports have been made on members of the *Gouania* genus. For instance, *G. longipetala* stem ethanolic extract was reported to contain reducing sugars, triterpenoids, phenolics, phytosterols, saponins and flavonoids, with total phenolic content of 52.02 mg/g tannic acid equivalent [16]. Crude extracts of *G. longipetala* leaves contained alkaloids, phenols, saponins, flavonoids, steroids, tannins, terpenoids and cardiac glycosides with concentrations of 34.30, 23.19, 18.10, 16.49, 13.74, 9.72, 7.45 and 3.89 mg/100 g, respectively. Furthermore, GC-MS analysis of *G. longipetala* leaf extract revealed that it had 20 compounds. These included spartein (constituting up to 24.92 %), 2-tetradecanol, dodecanoic acid, 1-octadecene, luparine, 5,6-dehydrolupanine, propanoic acid-3-chloro-methyl ester, sapogenine, catechin, flavon-3-ol, anthocyanin, resveratrol, linoelaidic acid, aragyrine, methyl-9,12-heptadecadienoate, baptifoline, oleic acid, spartein, kaempferol and ethyl oleate [78].

Different solvent extracts of *G. longispicata* were qualitatively screened for phytochemicals and reportedly had cardiac glycosides, phenolics, steroids, flavonoids, saponins, and resins [22]. Another study reported that phenols, tannins, alkaloids and resins were present in *G. longispicata* methanolic leaf extract [79], while the whole plant extract contained polyphenols, flavonoids, triterpenes, sterols and saponins [80]. Similarly, *G. longispicata* leaf extracts had flavonoids, cardiac glycosides, saponins, steroids, resins and phenolics [22]. In a quantitative study, petroleum ether and methanolic extract of *G. tiliaefolia* had total phenolic content of 78.30 mg GAE/g and 70.37 mg GAE/g [77]. Phytochemical analysis of *G. longipetala* leaf extracts quantified vitamin C (2.807 mg/100 g), phenolics (0.375 mg GAE/g) and flavonoids (11.615 μg/g), while the corresponding quantities in the stem extract were 2.126 mg/100 g, 0.299 mg GAE/g and 10.012 μg/g [81]. Some phytochemical studies have been done to isolate and characterize bioactive compounds from *Gouania* species. These are discussed in the following subsections.

### 3.1 Terpenoids from the genus *Gouania*

**Fig. 2:**
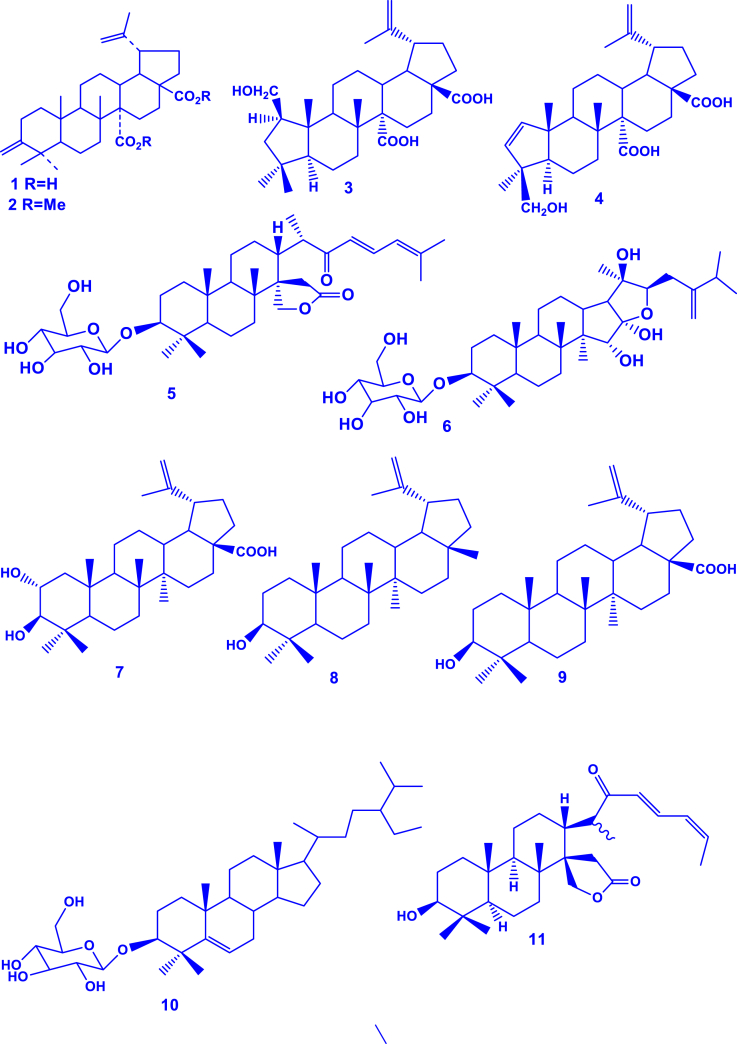

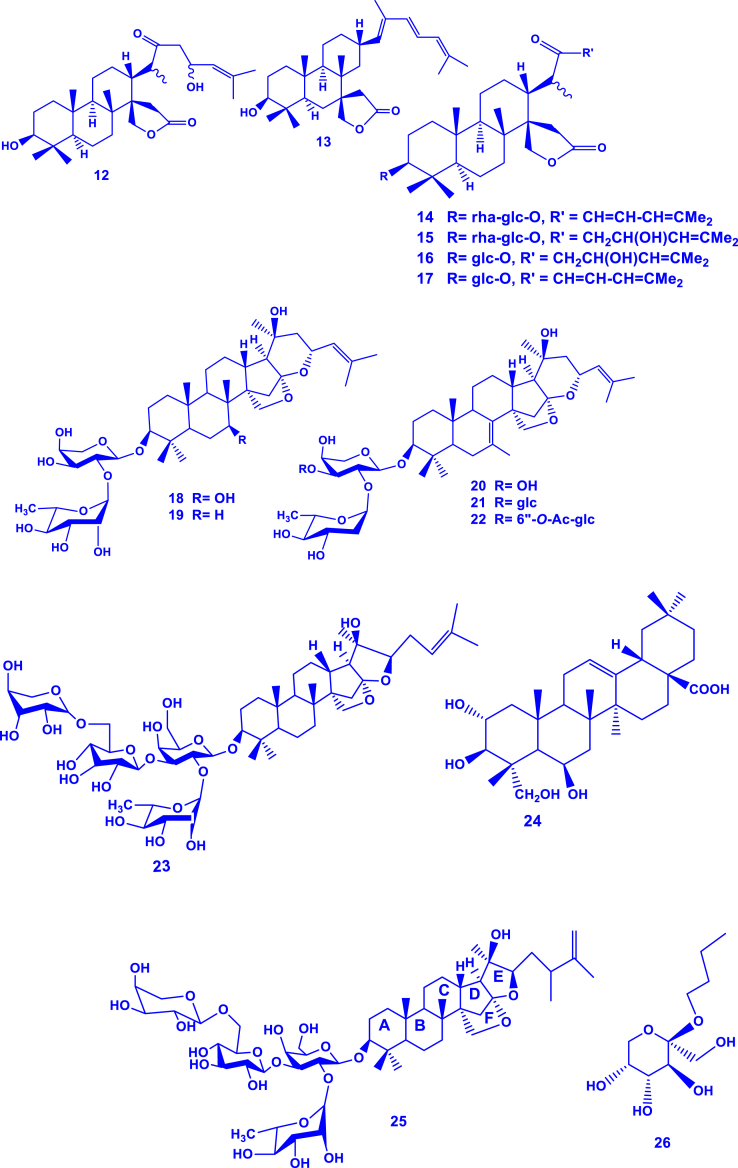

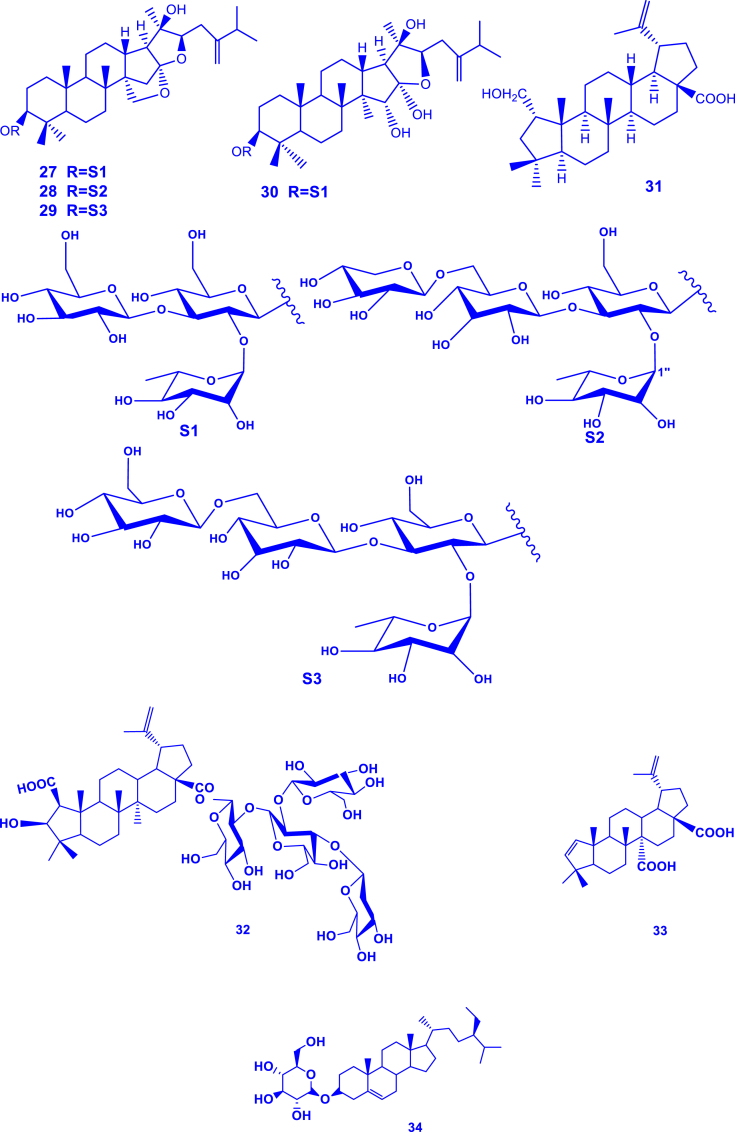
Terpenoids characterized in *Gouania* species (**1**–**34** correspond to the compounds in Table 2).

Terpenoids have been reported to dominate in this genus, with up to 34 of them reported (**1-34**) (Table 2; Fig. 2). For example, *G. lupuloides* stem yielded two novel terpenoids (gouanosides A and B) which are 16,17-seco-dammaranoid saponins [24]. A new triterpenic acid (gouanic acid) was also identified in *G. macrocarpa* leaf extract [13]. In another report, gouanic acid A and B were characterized in *G. ulmifolia* aerial part extracts [14]. Six new triterpenoid saponins (named gouaniaside I-VI) were characterized from *G. longipetala* aerial parts [25]. Later, four novel such saponins (named gouaniasides VII–IX), along with joazeiroside C were identified in *G. leptostachya* aerial parts [26]. A unique and novel ceanothane-type saponin was similarly characterized in *G. leptostachya* [82]. More recently, an alkyl glycoside (*n*-butyl-*β*-D-fructopyranoside) was characterized from *G. leptostachya* [82].

**Table 2:** Terpenoids reported from the genus *Gouania*.

Terpenoid	Species	Part used	References
Gouanic acid (**1**)	*G. microcarpa*	Leaves	[13]
Tetratriacontanoic acid (**2**)			
Gouanic acid A (**3**)	*G. ulmifolia*	Aerial parts	[14]
Gouanic acid B (**4**)			[14,27]
3-*O-β*-D-glucopyranosyl Gouanogenin A (**5**)	*G. longipetala*		[27]
Joazeiroside A (**6**)	*G. longipetala*, *G. leptostachya*		[1,27]
Alphitolic acid (**7**)			[25,27,82]
Lupeol (**8**)			[27,82]
Betulinic acid (**9**)	*G. longipetala*		[27]
*β*-sitosterol-3-*O- β*-D-glucoside (**10**)			
Gouanogenin A (**11**)	*G. lupulozdes*	Stem	[24]
Gouanogenin B (**12**)			
Ebelin lactone (**13**)			
Gouanoside A (**14**)			
Gouanoside B (**15**)			
Progouanogenin (**16**)			
3-*O-β*-D-glucopyranosyl gouanogenin A (**17**)			
Gouaniaside I (**18**)	*G. longipetala*	Aerial parts	[25]
Jujuboside I (**19**)			
Gouaniaside II (2**0**)			
Gouaniaside III (**21**)			
Gouaniaside IV (**22**)			
Gouaniaside V (**23**)			
Terminolic acid (**24**)			
Gouaniaside VI (**25**)			
*n*-butyl-*β*-D-fructopyranoside (**26**)	*G. leptostachya*		[82]
Gouaniaside VII (**27**)	*G. leptostachya*		[26]
Gouaniaside VIII (**28**)			
Gouaniaside IX (**29**)			
Joazeiroside C (**30**)			
Epigouanic acid A (**31**)			
Gouanioside A (**32**)	*G. leptostachya*		[82]
Ceanothenic acid (**33**)			
Daucosterol (**34**)

### 3.2 Phenolic compounds from genus *Gouania*

**Fig. 3:**
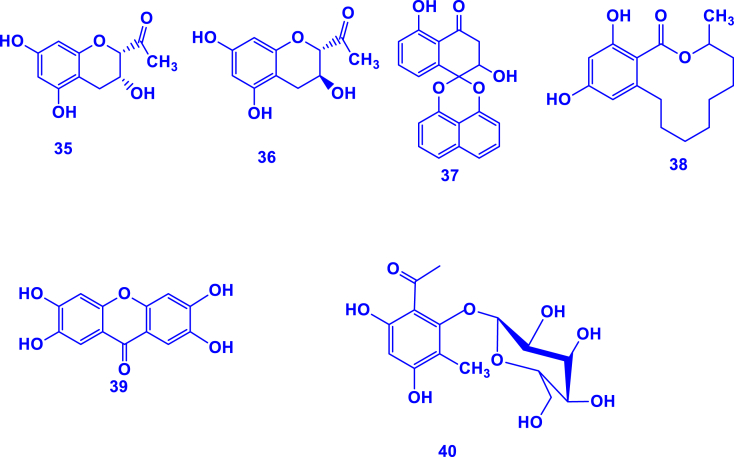
Phenolic compounds characterized in *Gouania* species (**35**–**40** correspond to the compounds in Table 3).

A total of five phenolic compounds (**35**–**40**) have been reported in *Gouania* genus (Table 3; Fig. 3). Mangiferin (**39)**, a well-known glucosylxanthone (xanthonoid) is the most important phenolic compound in this genus that was isolated from *G. obtusifolia* leaves [28]. This natural phenolic xanthonoid is known to possess excellent bioactivities such as antibacterial and anticancer activities [83]. A phenolic glycoside (named 4,6-dihydroxy-3-methylacetophenone-2-O-β-D-glucopyranoside (**40**)) was recently characterized from *G. longispicata* leaves [84].

**Table 3:** Phenolic compounds isolated from different parts of species in *Gouania* genus.

Phenolic compound	Species	Part	Reference
1-[(rel 2S,3R)-3,5,7-trihydroxy-3,4-dihydro-2H-chromen-2-yl]ethenone (**35)**	*G. leptostachya*	Stem	[85]
1-[(rel 2S,3S)-3,5,7-trihydroxy-3,4-dihydro-2H-chromen-2-yl]ethenone (**36)**			
Palmarumycin BG1/JC2 (**37)**	*G. longipetala*	Aerial parts	[86]
de-*O*-methyllasiodiplodin (**38)**			
Mangiferin (**39)**	*G. obtusifolia*	Leaves	[28]
4,6-dihydroxy-3-methylacetophenone-2-O-β-D-glucopyranoside (**40**)	*G. longispicata*	Leaves	[84]

### 3.3 Flavonoids from *Gouania* species

**Fig. 4:**
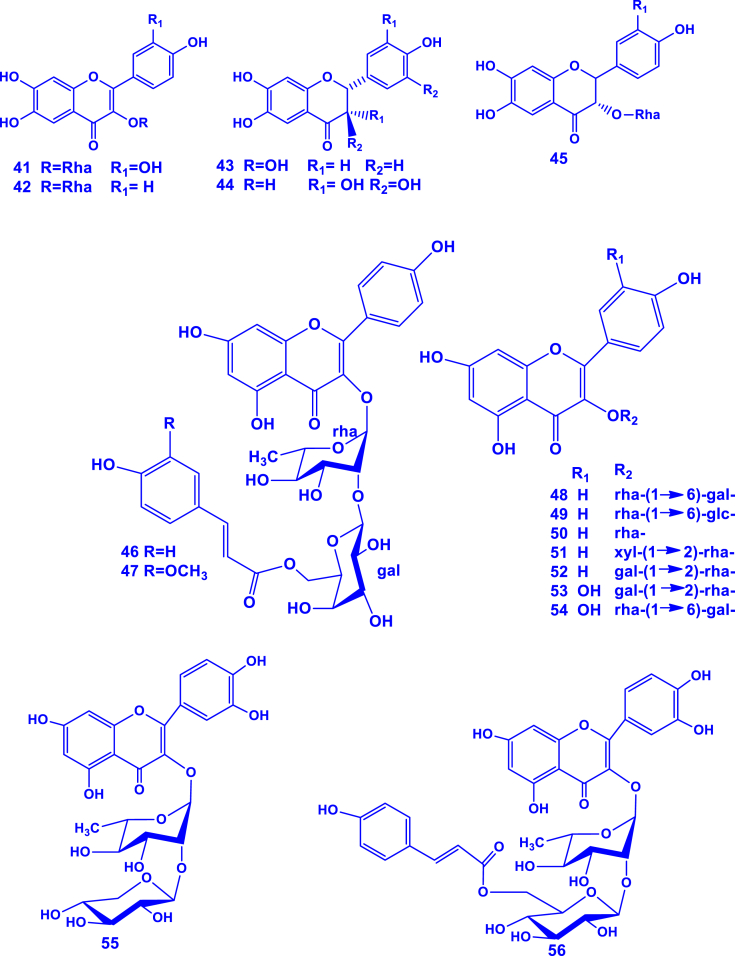

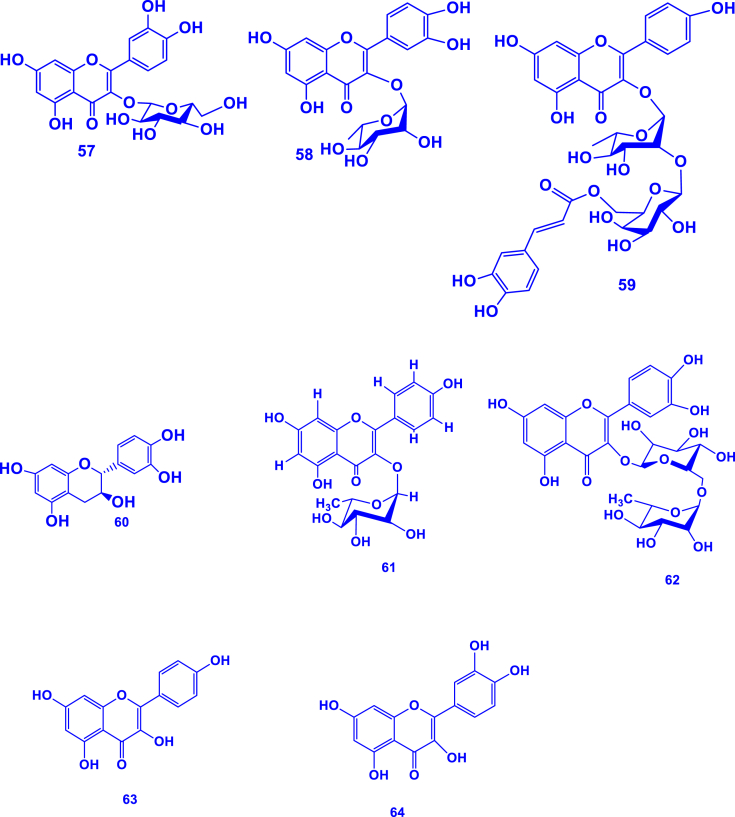
Structure of flavonoids isolated and characterized in the *Gouania* taxon. The numbers **41**–**64** refer to the molecules mentioned in Table 4.

To date, it is known that *Gouania* species contain phenolic compounds, either as phenolics or flavonoids [28,29], signifying their prevalence in this genus. A total of 24 flavonoids (**41**–**64**) have been reported in this genus (Table 4; Fig. 4). These include rutin (quercetin-3-O-rutinoside), kaempferol, quercetin and their derivatives. Such flavonoids play essential roles in plant defense mechanisms, contributing to protection against ultraviolet visible radiation, pathogens, and herbivores [87].

**Table 4:** Flavonoids isolated from the *Gouania* genus.

Flavonoids	Species	Part	Reference
Quercetin 3-*O*-α-L-rhamnoside (**41**)	*G. obtusifolia*	Leaves	[28]
Kaempferol 3-*O*-α-L-rhamnoside (**42**)			
Epicatechin (**43**)			
Gallocatechin (**44**)			
Engeletin (**45**)			
Kaempferol-3-*O*-(6-*O*-E-coumaroyl)-*β*-D-galactopyranosyl-(1→2)-α-L-rhamnopyranoside (**46**)	*G. longipetala*	Aerial parts	[29]
Kaempferol-3-*O*-(6-*O*-E-feruloyl)-*β*-D-galactopyranosyl-(1→2)-α-L-rhamnopyranoside (**47**)			
Kaempferol- 3-*O*-α-L-rhamnopyranosyl-(1→6)-*β*-D-galactopyranoside (**48**)			
Kaempferol-3-*O*-α-L-rhamnopyranosyl-(1→6)-*β*-D-glucopyranoside (**49**)			
Kaempferol-3-*O*-α-L-rhamnopyranoside (**50**)			
Kaempferol-3-*O*-*β*-D-xylopyranosyl-(1→2)-α-L-rhamnopyranoside (**51**)			
Kaempferol-3-*O*-*β*-D-galactopyr- anosyl-(1→2)-α-L-rhamnopyranoside (**52**)			
Quercetin-3-*O*-*β*-D-galactopyranosyl-(1→2)-α-L-rhamnopyranoside (**53**)			
Quercetin-3-*O*-α- L-rhamnopyranosyl-(1→6)-*β*-D-galactopyranoside (**54**)			
Quercetin-3-*O*-*β*-D-xylopyranosyl-(1→2)- -α- L- rhamnopyranoside (**55**)	*G. leptostachya*	Leaves	[1]
Quercetin-3-*O*-6-E-p-coumaroyl-*β*-D-glucopyranosyl-(1→2)-α-L-rhamnopyranoside (**56**)			
Quercitrin (**57)**	*G. leptostachya*	Aerial parts	[82]
Isoquercitrin (**58)**			
Kaempferol-3-*O*-(6-*O*-*E*-caffeoyl)-*β*-D-galactopyranosyl-(1→2)-*α*-L-rhamnopyranoside (**59)**			
Catechin (**60)**			
Kaempferol-3-O-α-rhamnopyranoside (**61**)	*G. longispicata*	Leaves	[84]
Rutin (**62**)			
Kaempferol (**63**)			
Quercetin (**64**)

Verily, alkaloids have been phytochemically identified among the secondary metabolites in *G. javanica* [46], *G. longipetala* [88], and *G. longispicata* [79]. However, no alkaloid has been isolated as a pure compound in any species of the *Gouania* genus. Therefore, future research could probe into the exploration and isolation of alkaloids within this genus. Despite the extensive traditional use of *Gouania* species reported in literature, characterization of bioactive compounds has been perfected in extracts from only 7 species (*G. longipetala*, *G. leptostachya*, *G. obtusifolia*, *G. macrocarpa*, *G. ulmifolia*, *G. longispicata* and *G. lupulozdes*). Specifically, *G. longipetala* has received the most attention in herbal medicine due to its widespread distribution compared to other *Gouania* species which grow in specific regions. Remarkably, more than 54 % of the yet characterized compounds from the *Gouania* genus are novel, suggesting its potential as a rich reservoir for discovering new drug leads to combat the growing array of pathogens. Therefore, extensive phytochemical investigations across a broader range of *Gouania* species beyond the seven investigated taxa could yield more compounds, which could act as scaffolds for development of potential drug candidates.

## 4 Bioactivity of extracts from *Gouania* species

### 4.1 Antioxidant activity

Ethanolic extract of *G. longipetala* stem had IC_50_ of 0.004 mg/mL [16]. The DPPH assay of methanolic leaf extract of *G. longipetala* showed that it exhibited antioxidant activity (16.6 %–65.5 %) [88]. In another study, *G. longipetala* extract produced a dose-dependent and significant increase in superoxide dismutase and decrease in malondiadehyde [18]. *G. longipetala* fresh leaf extract lowered malondialdehyde concentration significantly [78]. Petroleum ether extract of *G. tiliaefolia* had free radical scavenging activity with IC_50_ of 2.88 μg/mL, while the methanolic extract had IC_50_ of 4.79 μg/mL [77].

### 4.2 Antimicrobial properties

Leaf extracts of *G. javanica* showed antifungal activity against *Aspergillus niger*, *Candida albicans* and *Tricophyton mentagrophytes* with mean zone of inhibition of 21.16 mm, 12.08 mm and 8.55 mm, respectively [46]. Ethanolic extract of *G. longipetala* stems exhibited antibacterial effect against *Bacillus subtilis*, *B. thurigiensis*, *Staphylococcus aureus*, *Enterococcus faecalis*, *Proteus vulgaris*, *Pseudomonas aeruginosa*, *Salmonella typhi* and *Escherichia coli*, with the lowest MIC of 125 μg/mL against *B. subtilis* [16].

Recently, *G. longispicata* leaf extract inhibited growth of *S. aureus*, *Streptococcus pneumoniae*, *E. coli*, *P. aeruginosa*, *Candida albicans* and *Aspergillus flavus*, with *Streptococcus pneumoniae* being the most susceptible microorganism with MIC of 1.95 mg/mL and minimum bactericidal concentration of 15.625 mg/mL [22]. A similar study determined the antibacterial activity of nine Cameroonian medicinal plants against drug-resistant *E*. *coli*, *Enterobacter aerogenes*, *Klebsiella pneumoniae*, *Providencia stuartii*, *P. aeruginosa* and *S. aureus*, and the findings revealed that *G. longispicata* extract gave the broadest spectrum of action notably against 86.4 % of the bacterial strains tested [80]. Tannins, phenols, alkaloids and resins of *G. longispicata* were considered to be responsible for the potential activity against *S. aureus* and *Streptococcus pneumonia* and *P. aeruginosa* [79]. In another study, *G. lupuloides* was effective against *P. falciparum* K1 strain [39] and its stem extracts gave less than 50 % inhibition of *Mycobacterium tuberculosis* at 50 μg/mL [89].

### 4.3 Antidiabetic activity

Leaf extracts of *G. longipetala* significantly decreased the fasting blood sugar (FBS) levels of diabetic rats from 16.2 mM/L to 6.5 mM/L at 150 mg/kg within 24 h, and the FBS levels decreased by 62.0 %, 74.8 %, and 75.0 % on day 21 at 50 mg/kg, 100 mg/kg, and 150 mg/kg, respectively [88]. Using the oral glucose tolerance test, methanolic extract of *G. tiliaefolia* reduced blood glucose levels by 71.42 % and 75.39 % at doses 200 and 400 mg/kg-body weight, respectively compared with glibenclamide standard that reduced blood glucose levels by 66.17 % [77].

### 4.4 Anti-inflammatory activity

Ethanolic extract of *G. longipetala* stem bark had a maximal inhibitory effect of total edema by 93.78 % at 300 mg/kg [16]. A study assessing the anti-inflammatory effects of *G. leptostachya* extracts and fractions by inhibiting nitric oxide production in RAW 264.7 macrophages reported that the effects were concentration-dependent [26]. Another research group concluded that the ethanolic extract and fractions of *G. leptostachya* induced inflammatory effects by modulating nitric oxide synthesis in lipopolysaccharide-stimulated murine RAW264.7 macrophage cells, suggesting the potential therapeutic utility of *G. leptostachya* in inflammatory conditions [82].

### 4.5 Other bioactivities

There are other bioactivities of *Gouania* species. For example, the stem bark extract of *G. longipetala* has been shown to possess estrogenic properties [81]. Extracts of *G. tiliaefolia* showed insignificant thrombolytic and membrane stabilizing activities results compared to the respective standards [77]. Following castor oil-induced diarrhea, *G. tiliaefolia* methanol extract gave better reduction of diarrhea by 71.43 % (at 400 mg/kg-body weight) compared to loperamide (64.29 %) [66]. The central- and peripheral-analgesic activity of *G. tiliaefolia* methanol extract was dose-dependent compared to the standard, diclofenac sodium [77]. A study determined the anti-osteoclastogenesis of *G. javanica* leaf extracts and the results unveiled its potential as an effective inhibitor of osteoclastogenesis [90]. These components of *G. longipetala* exhibited various bioactivities including; inhibition of uric acid, amino acid decarboxylase activity, urine acidifiers, oligosaccharide provider, arachidonic acid inhibitor, decrease endothelial leukocyte and platelet adhesion [91].

## 5 Bioactivity of isolated compounds

Although 64 compounds have been characterized from *Gouania* genus, only 26 of these have been assessed for their bioactivities, such as antioxidant, anticancer, neuraminidase inhibition, and antibacterial properties (Table 5). Despite the documented isolation of compounds from this genus, scientific evaluation of efficacy, toxicity, and characterization of bioactive compounds has primarily focused on *G. longipetala*. Nevertheless, plants with significant ethnomedical usage have yielded potent compounds, with some integrated into essential medicines [92], which suggests that the genus *Gouania* can be a great source of new drug substances.

**Table 5:** Bioactivities of compounds isolated from genus *Gouania*.

Compound	Bioactivity tested	Species	Part used	Treatment/method	IC_50_ (μM)	References
Gouanic acid B (**4**)	Antioxidant	*G. longipetala*	Aerial parts	DPPH method	52.5	[27]
3-O-*β*-D-glucopyranosyl gouanogenin A (**5**)					56.4	
Joazeiroside A (**6**)					69.5	
Alphitolic acid (**7**)					68.2	
Lupeol (**8**)					63.2	
Quercetin 3-*O*-α-L-rhamnoside (**40**)	Neuraminidase inhibition	*G. obtusifolia*	Leaves	200 μg/mL (Neu5Ac2en control IC_50_ = 44.47)	7.89	[28]
Kaemferol 3-*O*-α-L-rhamnoside (**41**)					5.47	
Epicatechin (**42**)					5.49	
Gallocatechin (**43**)					10.22	
Engeletin (**44**)					5.44	
Mangiferin (**39**)					0.82	
4,6-dihydroxy-3-methylacetophenone-2-O-β-D-glucopyranoside (**40**)	Antioxidant	*G. longispicata*	Leaves	DPPH method	26.3	[84]
	Antibacterial			Broth dilution method	MIC = 32 μg/mL and 64 μg/mL	
Kaempferol-3-*O*-(6-*O*-E-feruloyl)-*β*-D-galactopyranosyl-(1→2)-α-L-rhamnopyranoside (**46**)	Antioxidant	*G. longipetala*	Aerial parts	100 mg/mL; Ascorbic acid control (IC_50_ = 60)	47.4	[29]
Kaempferol- 3-*O*-α-L-rhamnopyranosyl-(1→6)-*β*-D-galactopyranoside (**47**)	Antioxidant				40.4	
Quercetin-3-*O*-α- L-rhamnopyranosyl-(1→6)-*β*-D-galactopyranoside (**53**)					41.5	
Quercetin-3-*O*-*β*-D-galactopyranosyl-(1→2)-α-L-rhamnopyranoside (**52**)	Antioxidant	*G. longipetala*			13.8	
Jujuboside I (**19**)	Anticancer			HL60 cells	13.5	
Alphitolic acid (**7**)	Antibacterial activity			*S. aureus,* *E. faecalis* and *E. coli* (Gentamicin control MIC = 0.5)	MIC = 32, 64 and 128 mg/mL, respectively	
Gouaniaside VII (**27**)	Anti-inflammatory	*G. leptostachya*		NO inhibition by RAW 264.7 macrophages	The level of NO produced decreased in a concentration-dependent manner	[26]
Gouaniaside VIII (**28**)						
Gouaniaside IX (**29**)						
Joazeiroside C (**30**)						
Gouanioside A (**32**)	Anti-inflammatory	*G. leptostachya*		NO inhibition by RAW 264.7 macrophages	The level of NO produced decreased in a concentration-dependent manner	[82]
Kaempferol-3-O-α-rhamnopyranoside (**61**)	Antioxidant	*G. longispicata*	Leaves	DPPH method	20.0	[84]
	Antibacterial			Broth dilution method	MIC = 16 μg/mL and 32 μg/mL	
Rutin (**62**)	Antioxidant			DPPH method	28.1	
	Antibacterial			Broth dilution method	MIC = 16 μg/mL and 32 μg/mL	
Kaempferol (**63**)	Antioxidant			DPPH method	19.8	
	Antibacterial			Broth dilution method	MIC = 32 μg/mL and 125 μg/mL	
Quercetin (**64**)	Antioxidant			DPPH method	18.6	
	Antibacterial			Broth dilution method	MIC = 64 μg/mL and 32 μg/mL	

Note: IC_50_ values are in μM. MIC, Minimum inhibitory concentration.

Among the ten (10) compounds from *G. longipetala* tested for effects on promyelocytic leukemia HL60 and human erythromyeloblastoid leukemia K562 cell lines, only jujuboside I demonstrated moderate cytotoxicity [25]. Additionally, the antibacterial activity of the isolated compounds against S*taphylococcus aureus*, *Escherichia coli*, and *Enterococcus faecalis* revealed alphitolic acid as the sole compound with significant inhibitory activity against all the three bacteria, giving MIC values ranging from 32 to 128 mg/mL [25]. The first study of bioactive compounds from *G. longispicata* [84], found that compounds from its leaves methanolic extract (**40**, **61**–**64**) exhibited antibacterial activity against *S. pneumoniae* and *E. coli* with MIC from 16 to 125 μg/mL. The radical scavenging activities of compounds (**40**, **61**–**64**) from *G. longispicata* leaves had IC_50_ between 18.6 μg/mL and 28.1 μg/mL. The IC_50_ of **63** and **64** were not significantly different from that of ascorbic acid, which indicated their promising utilization as natural antioxidants.

## 6 Toxicity profile of *Gouania* species

Few toxicity studies have been reported for the genus *Gouania*. Acute toxicity tests of *G. longipetala* extract suggested no observable signs of toxicity or morbidity, with LD_50_ greater than 4000 mg/kg [88], while a sub-chronic study showed that the relative liver weight increased significantly on day 90 at 10 mg/kg [18]. Additionally, changes in hematological parameters were not significant for *G. longipetala* extract-treated and untreated rats, suggesting that the extract was safe [18]. However, long term therapy of *G. longipetala* (up to 90 days) suggested toxicity to the liver and kidney, particularly at high doses. Another report found that *G. longipetala* extract significantly reduced total cholesterol, triglycerides and very low-density lipoproteins, while high density lipoproteins and serum urea increased prominently on day 90 at 10 mg/kg. At all doses of *G. longipetala* extract tested, liver enzyme markers increased significantly [18]. Acute toxicity study of *G. longipetala* leaf extract revealed a LD_50_ > 5000 mg/kg bw, signifying safe oral use of the plant. Also, sub-acute administration did not significantly alter the hematological, hepatic and renal function indices [69]. The petroleum ether extract of *G. tiliaefolia* gave brine shrimp lethality LC_50_ = 2.59 μg/mL, while methanolic extract had LC_50_ of 3.38 μg/mL [77]. These findings imply that *Gouania* species are safe for traditional use, however, further studies need to be done to give scientific evidence of their safety.

## 7 Conclusions and future research directions

*Gouania* species are utilized traditionally for treatment of digestive, cardiovascular, respiratory, skin, musculoskeletal, reproductive, endocrine and urological systems ailments. Only seven *Gouania* species (*G. leptostachya*, *G. longipetala*, *G. macrocarpa*, *G. lupulozdes*, *G. ulmifolia*, *G. longispicata* and *G. obtusifolia*) have been investigated to date and found to exhibit anticancer, antimicrobial, antioxidant, and antiviral activities. In total, 64 compounds have been so far characterized from *Gouania* genus but studies targeting their toxicity are still limited. Future research on *Gouania* species should investigate the phytochemicals and bioactivities of the unstudied species such as *G. scandens*, *G. mauritiana*, *G. latifolia*, *G. polygama*, *G. tiliaefolia* and G. *meyenii* that have been indicated for use in herbal formularies. Given the sparse data on the toxicity profiles of *Gouania* species, there is a critical need for systematic toxicity studies to ensure their safety when used in herbal medicine. Additionally, conducting clinical studies to assess the efficacy and safety of *Gouania* extracts and the characterized bioactive molecules could provide valuable insights into their medicinal properties and support the discovery of novel bioactive molecules.

## CRediT authorship contribution statement

**Hannington Gumisiriza:** Writing – review & editing, Writing – original draft, Funding acquisition, Conceptualization. **Eunice Apio Olet:** Writing – review & editing, Supervision. **Lydia Mwikali:** Writing – review & editing. **Racheal Akatuhebwa:** Writing – review & editing, Data curation. **Owen Kembabazi:** Writing – review & editing. **Timothy Omara:** Writing – review & editing, Formal analysis. **Julius Bunny Lejju:** Writing – review & editing, Conceptualization.

## Data availability

This review generated no raw data.

## Funding

This review was funded by the Faculty of Science, 10.13039/501100009915Mbarara University of Science and Technology (Grant number: 7088522).

## Declaration of competing interest

The authors declare the following financial interests/personal relationships which may be considered as potential competing interests:Hannington Gumisiriza reports financial support was provided by 10.13039/501100009915Mbarara University of Science and Technology, Faculty of Science. Hannington Gumisiriza reports a relationship with 10.13039/501100009915Mbarara University of Science and Technology, Faculty of Science that includes: funding grants. All authors have no conflict. If there are other authors, they declare that they have no known competing financial interests or personal relationships that could have appeared to influence the work reported in this paper.
